# Temperature Gradient Effect on Gas Discrimination Power of a Metal-Oxide Thin-Film Sensor Microarray

**Published:** 2004-05-18

**Authors:** Victor V. Sysoev, Ilya Kiselev, Markus Frietsch, Joachim Goschnick

**Affiliations:** 1 Department of Physics, Saratov State Technical University, Polytechnicheskaya 77, Saratov 410054, Russia; 2 Institute for Instrumental Analysis, Forschungszentrum Karlsruhe, Hermann-von-Helmholtz-Platz 1, 76344 Eggenstein-Leopoldshafen, Germany

**Keywords:** Electronic nose, gas-sensor microarray, metal-oxide thin film

## Abstract

The paper presents results concerning the effect of spatial inhomogeneous operating temperature on the gas discrimination power of a gas-sensor microarray, with the latter based on a thin SnO_2_ film employed in the KAMINA electronic nose. Three different temperature distributions over the substrate are discussed: a nearly homogeneous one and two temperature gradients, equal to approx. 3.3 °C/mm and 6.7 °C/mm, applied across the sensor elements (segments) of the array. The gas discrimination power of the microarray is judged by using the Mahalanobis distance in the LDA (Linear Discrimination Analysis) coordinate system between the data clusters obtained by the response of the microarray to four target vapors: ethanol, acetone, propanol and ammonia. It is shown that the application of a temperature gradient increases the gas discrimination power of the microarray by up to 35 %.

## Introduction

There is a growing demand for simple instruments capable of classifying complex gas ensembles that odors or flavors usually are [1]. There are many applications for electronic olfaction in everyday life and also in industry including food product quality control, safety and security, environmental monitoring, indoor air quality, health care, medical diagnosis, pharmaceutical purposes, etc. [2]. However, recently there has been an increasing awareness of the fact that an electronic nose could be a universal tool of chemical gas analysis, no matter whether the gas components can be perceived as well by a human nose or not [3].

The development of e-nose systems primarily focuses on the study of arrays of gas sensors of various operation principles [2-4]. The complexity, large size, and high cost of these instruments considerably restrict possible fields of application. However, progress in microsystem technology has made it possible to design e-noses on a chip basis (for example, [5-7]). Such instruments offer obvious advantages over conventional electronic noses with macrostructures: lower costs, lower power consumption, small size, etc. It seems that only microsystem platforms are able to meet the demand for the future market of electronic noses.

At present, most of the commercial electronic nose systems are still employing gas sensors produced separately and mounted individually on a bulky carrier. This, however, makes production time-consuming and therefore, costly. Moreover, the use of different sensor materials causes individual aging of the sensor elements, which inherently interferes with the long-term stability of the signal patterns used for gas-analysis. Similarly, separate gas sensor pads of different materials are applied in usual microsystems to realize a gas sensor array. Thus, the same drawbacks occur due to the use of different sensing materials: individual aging as well as cost-intensive deposition.

In contrast, the KAMINA, a microsystem electronic nose developed at the Karlsruhe Research Center, utilizes a single metal-oxide thin film segmented by electrodes to create a gas sensor microarray [7,8]. It employs two different techniques, a temperature gradient and surface filters to modify the properties of the sensor segments in a controlled manner [8]. This sensor-array design perfectly meets the mentioned requirements for low-cost high-volume production in combination with high gas analytical performance.

In the present contribution, the effect of the spatial variation of the operating temperature applied across the segmented metal-oxide thin film is thoroughly studied in order to improve the gas discrimination power of the KAMINA system. The investigation is based on the signal patterns obtained for 4 model gases: ammonia, ethanol, acetone and propanol.

The gas-discrimination power of the device is judged by using Linear Discriminant Analysis (LDA), an effective tool to determine the differences of the signal patterns obtained from the microarray as a result of the exposure to the vapors of interest [9,10].

## Experimental

### The KAMINA gas sensor microarray (GSMA)

**Figure 1.**
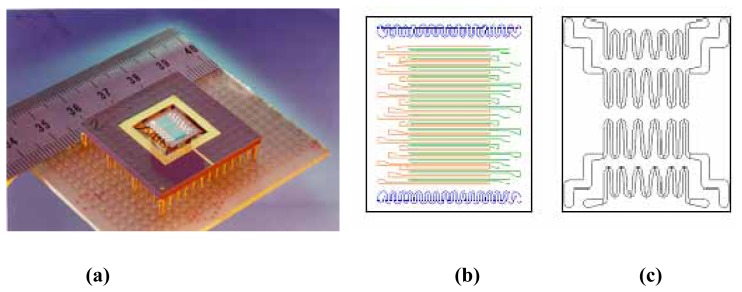
(a) - The photo of GSMA used in this study, (b) front-side scheme of the chip: the two thermoresistors (blue), the Pt electrodes for SSs (green and red), (c) backside of the chip carrying the heaters.

The fabrication of the GSMA used in this study is performed in four steps. The first step is the deposition of the gas-sensitive SnO_2_:Pt film by r.f. magnetron sputtering using a shadow mask. The target is fabricated of pressed SnO_2_ powder (99.9 % purity, Cerac Corp., USA). In the center of the target a cylindrical, core of platinum (99.95% purity, Leybold Materials GmbH, Germany) with a diameter of 5.5 mm is incorporated in order to achieve doping of the SnO_2_ films with a platinum content of 0.8 at.%. The sputter gas is a 4:1 Ar/O_2_ mixture. The substrate used is a Si wafer of a 3-inches diameter, thermally oxidized on both sides. The used sputter technique allows the fabrication of homogeneous gas-sensitive films [7,8]. The as-deposited films are annealed under a flow of clean air at a temperature of 600 °C for 5 hours to improve the morphology of the layer. In the next step, the Pt strip electrodes and two meander-shaped thermoresistors with a thickness of approx. 1 μm are sputter-deposited on the same side of the substrate as the SnO_2_ film, under a shadow mask for structuring the films. The arrangement of the electrodes as shown in Figure 1(b) subdivides the monolithic SnO_2_ film into 38 sensor segments (SS), on an area of 4×8mm^2^. In the third step, platinum heaters are deposited onto the backside of the substrate structured as in Figure 1(c). The thickness of the heaters is adjusted to be approx. 1μm, resulting in a resistance of the heaters of a few Ohms. 3″ Si wafers and 6″ Si wafers are used carrying 26 or 122 GSMAs, respectively. After dicing the wafer, each GSMA of the batch is mounted within a 120-pin carrier (PGA-120, Kyocera Co., Japan) on ceramic tubes (Figure 1(a)). In order to reinforce the temperature gradient, only one side of the GSMA is fixed by the tubes using ceramic glue (Resbond 940 adhesive, Cotronics Co., USA). The electrical connections of the electrodes, thermoresistors and heaters are made via ultrasonic bonding with gold wires (Wedge-Wedge Wire Bonder 7400B, West Bond Inc., USA).

### Set-up, measuring procedure and data processing

**Figure 2.**
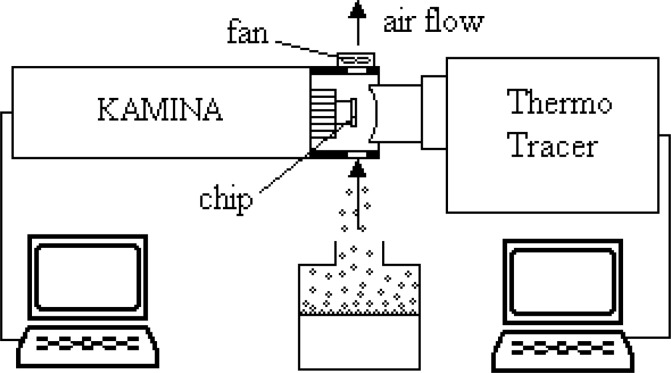
Scheme of the experimental set-up.

The results presented in this paper have been obtained by means of the set-up schematically shown in Figure 2. The set-up simulates the conditions of open-air applications of an e-nose.

The KAMINA was connected through a RS232 interface with a PC and operated by the MINOS (Micronose Operating System) software. The latter allows setting the measuring conditions of the KAMINA and it visualizes the recorded data or results of the signal pattern analysis applying multivariate data processing methods. MINOS was especially used for adjusting the power consumption of the GSMA heaters and thus, for setting the necessary inhomogeneous heating of the substrate. The temperature distribution across the chip is controlled via two Pt thermoresistors placed at the front side of the chip. The SS resistances were measured between two adjacent electrodes. The data were monitored and stored by a computer.

The infrared Thermo Tracer TH3100MR (NEC San-ei Instrum. Ltd, Japan), connected via DIF board with a computer, was applied to observe the temperature distribution *in-situ* across the GSMA surface exposed to gases. The emission level of the IR tracer has been calibrated according to data obtained on similar samples.

The sensor has been exposed to four vapors extracted from ethanol, acetone, propanol and ammonia liquids. An open small reservoir of the liquids was placed in the airflow forced by the fan integrated in the head cover of the KAMINA module as shown in Fig. 2.

**Figure 3.**
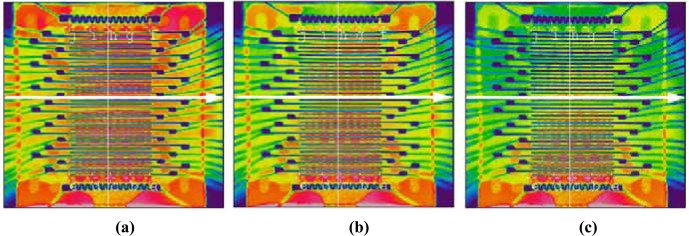
The infrared images of the heated GSMA surface recorded under application, no *gradT* (a), *gradT* of 3.3. °C/mm (b), *gradT* of 6.7 °C/mm (c). The white arrow shows the airflow direction over the GSMA.

In order to investigate the effect of the spatial temperature variation on the gas-recognition power of the GSMA, three temperature regimes were applied to the microarray (Figure 3):

1. The heaters' power input was adjusted to homogenize the temperature distribution on the chip measured with the thermoresistors at the chip edges. A temperature of approx. 360 °C was tried to be maintained. Some measurements were performed at other temperatures in the range of 295-400 °C.
2. The temperature gradient (hereafter, *gradT*) of 6.7 °C/mm was applied across the SSs, resulting in a temperature difference of approx. 50 °C across the array. The minimum temperature was about 310 °C.
3. An intermediate *gradT* of 3.3 °C/mm was applied across the SSs. This regime corresponded to a temperature variation of approx. 25 °C. The minimum temperature was about 330 °C.

The mentioned range of operating temperatures was chosen as, on the one hand, the sensor response at temperatures below 300 °C becomes quite slow for real-time measurements, while, on the other hand, the gas sensitivity of the SSs sharply decreases above temperatures of 400 °C.

The GSMA was exposed to vapors in the sequence: clean air → ethanol → clean air → acetone → clean air → propanol → clean air → ammonia → clean air, etc. The “clean air” term in the study relates to conditions when the liquids of interest were removed from the airflow through the KAMINA head cover. The exposure time of the GSMA to vapors and clean air was set to about one minute. The measuring period was adjusted to 1 sec. for a whole readout of the array.

**Figure 4.**
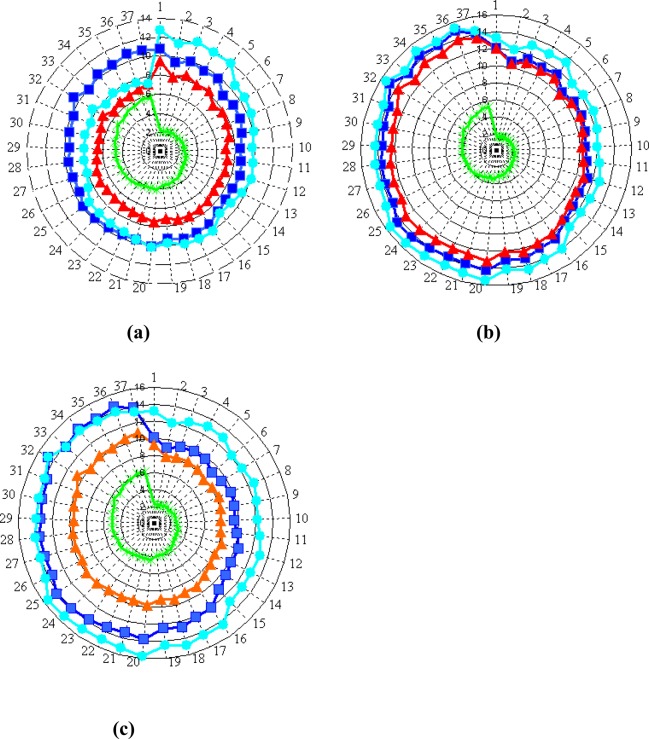
Radar plots of the 38 SS resistances of the GSMA operated with, no *gradT* (a), *gradT* of 3.3. °C/mm (b), *gradT* of 6.7 °C/mm (c). The raw resistance values are given in MOhm. The red, blue, green and light blue points show the resistance data at GSMA exposure to acetone, ethanol, ammonia, and propanol vapors.

The typical radar plots of the SS resistances caused by the presence of the vapors are shown in Figure 4. The resistance values are averaged for the time of GSMA exposure to the vapor.

To be treated by LDA, the raw SS resistance data were processed as

(1) $$R_{i}\to r_{i}=\log\left(\frac{R_{i}}{R_{med}}\right)$$

*R_i_* and *r_i_* are resistance and normalized resistance of the *i*-segment in the GSMA, respectively, *i*=[1,38]; *R_med_* is the median of all resistance data recorded during measurement. This normalization is applied to eliminate the dependence of the sensor resistances on the vapor concentration to a high extent. Only the steady-state values of the SS resistances were utilized for the LDA treatment.

## Results and Discussion

**Figure 5.**
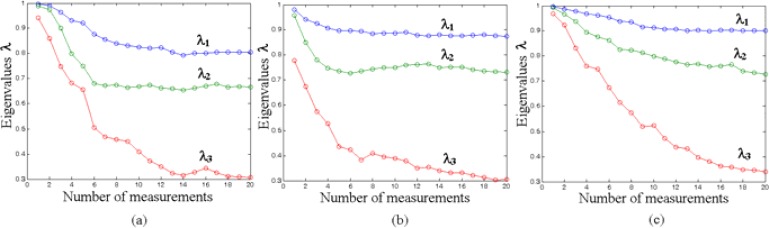
The dependence of eigenvalues, λ_1_-λ_3_, of the LDA model on the number of measurements. The model is based on data obtained under the three temperature regimes: no *gradT* (a), *gradT* of 3.3 °C/mm (b), and *gradT* of 6.7 °C/mm (c).

Like all supervised multivariate techniques, the LDA method needs a relatively high amount of training data. As an indication of sufficient training, the eigenvalues of the LDA model plotted versus the number of included experiments should come to stationary values, i.e. increasing the number of included measurements should not change the eigenvalues anymore. For validation, each follow-up measurement (GSMA exposure to all vapors in sequence) has been tested versus the LDA model developed with the earlier data. The results are summarized in Figure 5.

**Figure 6.**
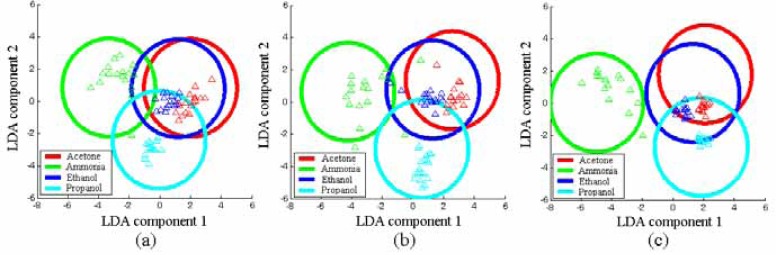
Testing results (points) of the final LDA model (ellipses). The confidence probability is 0.99 for the drawn ellipses.The plane of the first two LDA components is shown. The data are based on the GSMA operated with, no *gradT* (a), *gradT* of 3.3 °C/mm (b), *gradT* of 6.7 °C/mm (c).

Each point within the plot is a single GSMA exposure (performed over a number of days) to the complete sequence of all vapors. As can be seen, the LDA models for the three operating conditions finally became stationary enough to be used for reliable recognition. The testing showed a successfull recognition of target vapors in 85-96 % of the data sets. The percent of recognition was calculated as the ratio of data correctly predicted by LDA to all tested data obtained under the exposures. In general, the fraction of correct predictions is increased with growing *gradT* value. Figure 6 depicts the results of testing data of one of the last cycles of gas exposures.

It is noteworthy that the LDA models based on a high number of exposures show much smaller distances between the clusters and more scatters than the ones based on data from a single gas exposure. This is because the experiments were made under realistic practical conditions in which the uncertainties of the vapor formation and transport to the KAMINA chip contribute to the signals obtained. The results given in Figure 6 are independent from the chip exposure scatters. One of the interesting features in Fig. 6 is that growing of *gradT* forces the ellipses centers of the four vapor patterns to move away from the center of the LDA coordinate system. Hence, the increase of *gradT* causes the signal patterns of the four vapors to become more distinctive. To visualize this effect, let us define the gas discrimination power of the sensor array as the average distance of the gas-specific cluster centers, *D_avg_*, in the LDA coordinate system, known as a Mahalanobis distance, according to

(2) $$D_{avg}=\sqrt{\sum_{i}\frac{\lambda_{i}}{1-\lambda_{i}}}$$

Here, *λ_i_* are the eigenvalues (in our case, *i*=(1,3)).

**Figure 7.**
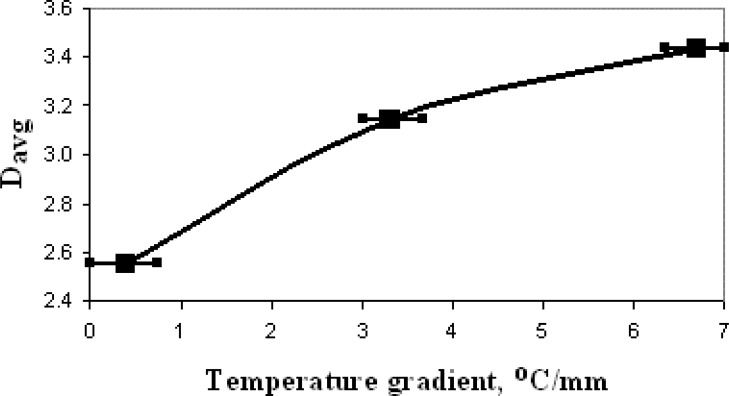
The dependence of the average distance of cluster centers in the LDA coordinate system, *D_avg_*, on the temperature gradient applied to GSMA.

This average distance, *D_avg_*, calculated by this way is depicted in Figure 7 as a function of *gradT* applied to GSMA.

Thus, the *D_avg_* obtained when the GSMA was operated with *gradT* of 6.7 °C/mm exceeds the *D_avg_* obtained without *gradT* by a factor of about 1.35. That means 26 % of the total discrimination for the tested gases is due to the temperature gradient.

**Figure 8.**
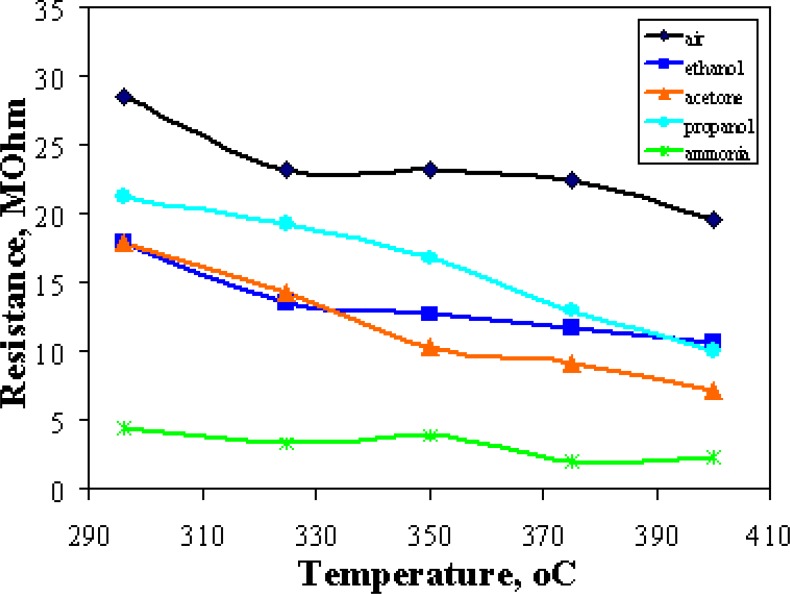
The median SS resistance of the GSMA *vs.* the operating temperature at different gas exposures. The data were recorded with the GSMA working under nearly homogeneous temperature conditions.

It shows that a discrimination of vapors with the GSMA is already possible without using *gradT,* but with lower validity. Probably, a slight scatter of the operating temperature and material or structural inconsistencies of the sensor by means of other factors may contribute to this effect [11]. Further work will be carried out to investigate the enhancement of gas discrimination by the temperature gradient in more detail. Figure 8 shows the gas-specific differences in the temperature dependence of the SS resistances. Due to the mostly falling resistances with increasing temperature, the resistance patterns become more pronounced. As the temperature dependence is characteristic of the type of vapor, the distinctiveness of the resistance patterns increases, as well, resulting in the observed enhanced discrimination power with growing temperature gradient. Accordingly, the effect of the temperature gradient is dependent on the type of gases the microarray is exposed to. Moreover, not only the span of temperature variation is important for gas discrimination, the average temperature on the chip is it, as well.

## Conclusions

The gas-sensor microarray (GSMA) of the Karlsruhe Micronose (KAMINA), based on a single metal-oxide film, applies a temperature gradient to reinforce the gas discrimination. The influence of the temperature gradient on the gas-discrimination power has been investigated. The response of the array operated under nearly homogeneous temperature conditions and the two temperature gradients of 3.3 °C/mm and 6.7 °C/mm, to ethanol, acetone, propanol and ammonia have been analyzed by Linear Discriminant Analysis. It has been shown that the gas discrimination power of the microarray, defined as a Mahalanobis distance in the LDA coordinate system, is increased by about 35 % with *gradT* at 6.7 °C/mm with respect to a homogeneous temperature distribution. The results prove that the inhomogeneity of the operating temperature contributes substantially to the gas discrimination power of the microarray. However, other material-dependent differences in the sensor segments of the microarray differentiate the selectivity of the microarray's sensor elements as well. It will be the subject of future investigations to generalize the results in order to further increase the positive effect of the temperature gradient on the gas-discrimination power of the KAMINA microarray.
